# Stigma and Social Determinants of Health Associated With Fidelity to Guideline-Concordant Therapy in Patients With Breast Cancer Living With and Without HIV in Botswana

**DOI:** 10.1093/oncolo/oyad183

**Published:** 2023-07-05

**Authors:** Yehoda M Martei, Modesty Obasohan, Lebogang Mokokwe, Tlotlo Ralefala, Mosepele Mosepele, Robert Gross, Frances K Barg

**Affiliations:** Department of Medicine (Hematology-Oncology), University of Pennsylvania, Philadelphia, PA, USA; Perelman School of Medicine, University of Pennsylvania, Philadelphia, PA, USA; Botswana University of Pennsylvania Partnership, Gaborone, Botswana; Faculty of Medicine, University of Botswana, Gaborone, Botswana; Princess Marina Hospital, Gaborone, Botswana; Faculty of Medicine, University of Botswana, Gaborone, Botswana; Department of Medicine (Infectious Diseases), University of Pennsylvania, Philadelphia, PA, USA; Department of Family Medicine and Community Health, University of Pennsylvania, Philadelphia, PA, USA

**Keywords:** breast cancer, HIV, health care disparities, treatment fidelity

## Abstract

**Background:**

Patients with breast cancer in sub-Saharan Africa (SSA) experience a disproportionate burden of mortality. Fidelity to treatment guidelines, defined as receiving optimal dose and frequency of prescribed treatments, improves survival. We sought to identify patient factors associated with treatment fidelity and how this may differ for people with HIV (PWH) and breast cancer.

**Methods:**

We conducted a qualitative study of women who initiated outpatient treatment for stages I-III breast cancer in Botswana, with deviance sampling of high- and low-fidelity patients. One-on-one interviews were conducted using semi-structured guides informed by the Theory of Planned Behavior. The sample size was determined by thematic saturation. Transcribed interviews were double coded with an integrated analytic approach.

**Results:**

We enrolled 15 high- and 15 low-fidelity participants from August 25, 2020 to December 15, 2020, including 10 PWH (4 high, 6 low fidelity). Ninety-three percent had stage III disease. Barriers to treatment fidelity included stigma, social determinants of health (SDOH), and health system barriers. Acceptance and de-stigmatization, peer and other social support, increased knowledge and self-efficacy were identified as facilitators. The COVID-19 pandemic amplified existing socioeconomic stressors. Unique barriers and facilitators identified by PWH included intersectional stigma, and HIV and cancer care integration, respectively.

**Conclusion:**

We identified multilevel modifiable patient and health system factors associated with fidelity. The facilitators provide opportunities for leveraging existing strengths within the Botswana context to design implementation strategies to increase treatment fidelity to guideline-concordant breast cancer therapy. However, PWH experienced unique barriers, suggesting that interventions to address fidelity may need to be tailored to specific comorbidities.

## Teaser

Fidelity to treatment guidelines, defined as receiving optimal dose and frequency of prescribed treatments, improves survival. This study aimed to identify patient factors associated with treatment fidelity and how this may differ for people with HIV and breast cancer.

**Implications for Practice**

Social determinants of health (SDOH), including breast cancer stigma, and health system factors may adversely impact initiation and adherence to guideline-concordant breast cancer treatment among patients with curative disease in Botswana and potentially other countries in sub-Saharan Africa (SSA). Understanding the prevalence of stigma and SDOH in this population is important for the prioritization and implementation of strategies to promote optimal levels of treatment adherence among patients with breast cancer in Botswana. People with HIV experienced unique barriers, suggesting that interventions to address treatment fidelity may need to be tailored to individual circumstances such as comorbidities.

## Background

Breast cancer mortality rates have declined in the US and other high-income countries in the last 40 years,^1^ partly due to improvements in evidence-based systemic therapies and other multimodality treatments.^1^ Sub-Saharan Africa (SSA) has the highest age-standardized breast cancer mortality rate globally,^2,3^ where the mortality to incidence ratio is 0.57 compared with 0.15 in countries in North America.^4,5^ In addition, SSA has 74% of the global population of women with HIV with a concurrent diagnosis of breast cancer.^6^ Recent studies have shown worse survival in people with HIV (PWH) who are diagnosed with breast cancer. A recent study from Botswana showed that PWH and breast cancer had an elevated risk of 2-year all-cause mortality compared to patients with breast cancer without HIV (HR = 2.68; 95% CI, 1.17-6.13).^7^

Reasons for lower breast cancer survival outcomes in SSA are likely multifactorial including patient and health system delays that lead to advanced stage at presentation,^8-10^ and more aggressive molecular subtypes (eg, “triple negative” breast cancer).^11^ Relative dose intensity is a composite measure of cumulative dose of chemotherapy received and duration of treatment received, expressed as a proportion of standard dose and duration and has been associated with survival outcomes.^12,13^ Few studies from SSA have also addressed the quality of care for patients with breast cancer.^14,15^ Notably, we showed that patients with breast cancer in Botswana received low relative dose intensity of chemotherapy, which was even lower for PWH, which may account for some of the suboptimal outcomes in this setting.^7^

Unlike most countries in SSA, Botswana’s Ministry of Health has developed evidence-based national breast cancer guidelines informed by specific diagnostic, medication, and personnel resources available in-country.^16,17^ Patients with breast cancer in Botswana may be treated with various combinations of surgery, radiation therapy and endocrine or targeted or cytotoxic systemic therapy. Patients receive chemotherapy in an outpatient infusion clinic and the Ministry of Health allocates funding for the purchase of all drugs on the national essential medicines list. However, survival outcomes will only be improved if there is adherence to guideline-concordant therapy.^18,19^ In a recent study, we identified health system (including drug stockouts),^20^ and provider barriers to “treatment fidelity”, defined as the extent to which guideline-concordant therapy is delivered as planned.^21,22^ Assessing treatment fidelity is necessary for understanding how fidelity acts as a potential moderator of the relationship between interventions and intended outcomes,^23^ and whether the failure of these guidelines is attributable to poor or inadequate multilevel implementation,^24^ or other unrelated factors.^22,25^ We aimed to evaluate patient-reported socioeconomic and cultural factors associated with adherence to guideline-concordant breast cancer therapy as planned, and how this may differ for PWH.^25^

## Methods

### Theoretical Framework

**Figure 1.**
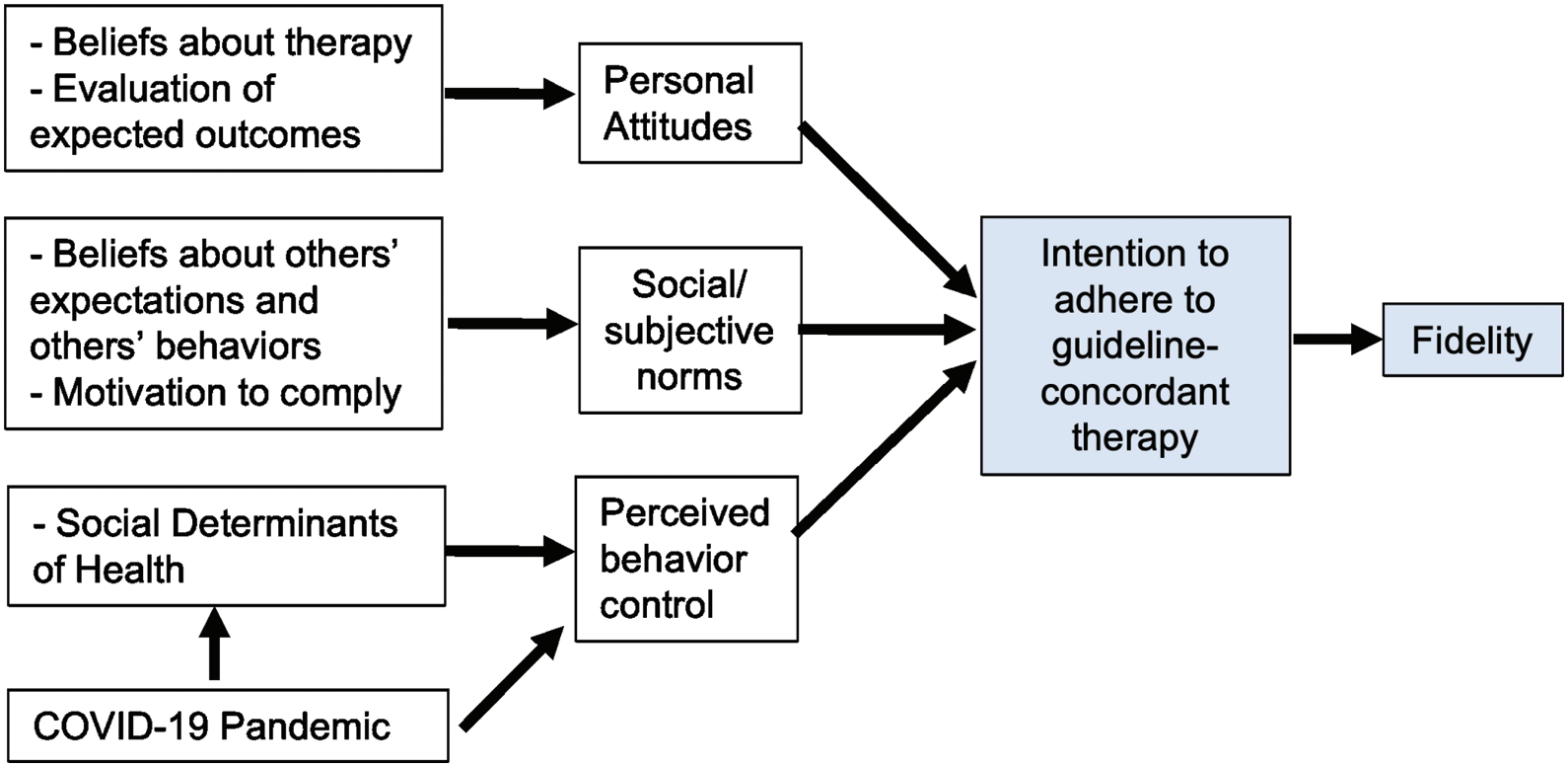
Modified Theory of Planned Behavior, adapted for fidelity to guideline-concordant therapy.

We chose the Theory of Planned Behavior (TPB) because it reflects the most empirically predictive causal pathways of decision making and behavior change (Fig. 1).^26^ The TPB holds that intentions play an important role as the most proximal determinant of behaviors. Intentions, deﬁned as a person’s motivation—or effort the individual plans to exert—to perform a behavior, are a necessary precursor for behavior to occur.^27^ Relatively strong intentions will lead to change in behavior if the individual has the resources needed to perform the given behavior.^27,28^ The framework examines personal attitudes, that is positive or negative beliefs about the behavior, and perceived behavioral control, the extent to which the behavior is perceived as easy to be done.^29^ Importantly, it also evaluates subjective norms, which refers to a person’s beliefs about whether peers and people of importance to the person think they should engage in the behavior.^27,28,30^ Subjective norms also consider perceived social norms which refers to customary codes of people in a group or people or larger cultural context.^26,31-35^ The TPB has been used and validated for understanding various behaviors related to health promotion in non-communicable diseases, for example, diabetes self-care,^36,37^ mammography,^38^ and cervical cancer screening.^29,38-40^

### Patient Selection

We used a deviance sampling^40^ approach to recruit up to 30 participants with breast cancer who had completed systemic treatment at Princess Marina Hospital in Gaborone. These included participants who had also completed other multimodality treatment, including surgery and/or radiation therapy at the time of enrollment. Deviant case sampling utilizes purposive sampling of extreme cases of a phenomenon—in this instance, we used high vs low fidelity.^40^ We also purposively sampled PWH in both high- and low-fidelity groups. We defined high (*n* = 15) and low (*n* = 15) fidelity to guideline-concordant therapy based on relative dose intensive of chemotherapy ≥0.85 vs <0.85, based on prior work.^12^ Although the ascertainment of relative dose intensity accounts for treatment delays, it does not differentiate between patient-initiated vs provider-initiated delays. The sample size was assessed to fall within an acceptable range for qualitative study design^41^ and was further confirmed based on thematic saturation. Participation in the study was voluntary and patients were offered the option of written and verbal informed consents because of the potentially sensitive nature of the interviews.

### Data Collection and Analysis

We used one-on-one open-ended qualitative interviews for this study to maximize privacy in the discussion of possibly stigmatizing beliefs and behavior, and to be able to link individual characteristics to participants’ ideas.^42^ Trained female Motswana staff conducted the interviews, to maximize cultural, gender and language-matching to study participants,^43^ and to encourage respondents to respond completely and honestly.^44^ A draft interview guide was developed based on the TPB framework.^45^ The interview guide was modified through an iterative process based on emerging themes. Each interview was conducted via phone and audio recorded in Setswana (the national language in Botswana), transcribed verbatim, and translated to English. Some interviews were a mix of Setswana and English. A sample of interviews were double transcribed and back translated to Setswana to assess accuracy of translations. Inconsistencies were resolved through repeat audio review and group discussions. Transcribed interviews were imported into NVivo 12.0 (QSR International, Melbourne, Australia) for coding and analysis. We used an integrated approach to the analysis.^46^ We developed an initial codebook based on constructs from the TPB and incorporated new codes that emerged from the data. Each code was defined, including decision rules and examples. Each interview content was double coded, allowing for multiple themes and discordant codes were discussed with a qualitative expert, until a minimum of 80% agreement was reached for all codes. We used the attribute function to assign fidelity and HIV status in our analysis. Once all data were coded, we examined each code for patterns and themes to ultimately form a theory about the data.

### Ethical Clearance

The University of Botswana, Health Research Development Committee–Ministry of Health and the University of Pennsylvania Institutional Review Boards approved the study.

## Results

### Participant Characteristics

We approached 33 women for enrollment, and 94% and 88% of the high- and low-fidelity participants, respectively, consented. Participant characteristics are summarized in Table 1. Ten participants (33%) (4 high, 6 low fidelity) were PWH.

**Table 1.** Summary of participants characteristics grouped by high vs low fidelity.

	High fidelity	%	Low fidelity	%
Age				
<50	7	47	6	40
≥50	8	53	9	60
Hormone receptor status				
Positive	8	53	8	53
Negative	7	47	4	27
Unknown	0	0	3	20
HER2 status				
Positive	4	27	5	33
Negative	11	73	9	60
Unknown	0	0	1	7
Cancer stage				
II	1	7	7	47
III	14	93	8	53
HIV status				
Positive	4	27	6	40
Negative	11	73	9	60
Intent of chemotherapy				
Adjuvant	3	20	7	47
Neoadjuvant	12	80	8	53

### Summary of Findings

**Figure 2.**
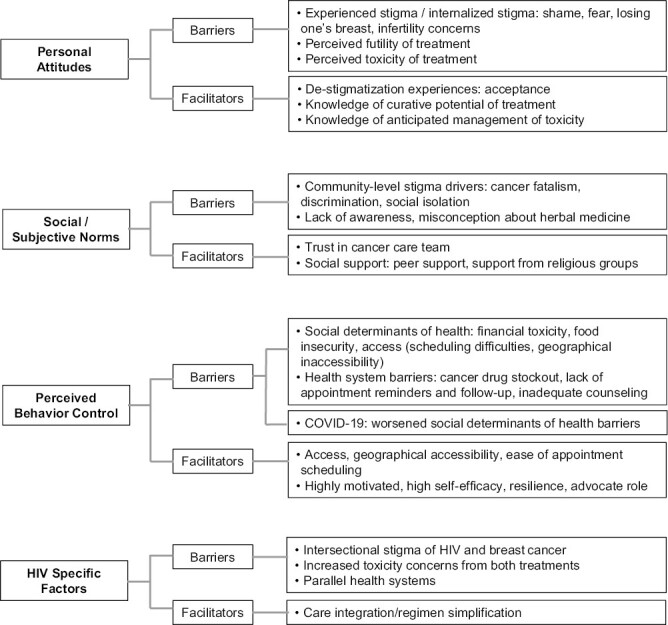
Coding tree summarizing barriers and facilitators within the theory of planned behavior domains and specific HIV factors.

Figure 2 provides a summary of the barriers and facilitators identified and how they map on different domains of the TPB. There were no barriers or facilitators exclusively identified in the high- or low-fidelity group. Barriers and facilitators specific to PWH were summarized separately in Fig. 2.

### Personal Attitudes: Barriers and Facilitators

#### Breast Cancer Stigma

Study participants identified the experience of stigma and internalized stigma as barriers that led to delays in initiating treatment, treatment abandonment during systemic treatment and forgoing a mastectomy in some cases. Stigma-related experiences included fear associated with the diagnosis of cancer and death from cancer, shame related to appearance and the risk of “disfigurement or disability” for women losing one or both breasts following a mastectomy, and the perceived risk of infertility following chemotherapy:

*the first thing that came to my mind [after cancer diagnosis] was that… my time to die was quite near... I dragged my feet when it came to removing the breast… I lapsed for about six to eight months before I went for surgery because I was asking myself what the point of removing the breast was if I was still going to die. (Low fidelity, HIV-)*

#### Futility and Toxicity Related to Cancer Treatment

The study participants identified perceived treatment futility and concerns about treatment toxicity as barriers that either led to delays during treatment or early discontinuation of treatment. They also identified lack of knowledge about the intent of the cancer-directed therapies as a barrier to receipt of care. They ascribed their lack of knowledge to limited information received from the cancer care team. Participants also reported limited knowledge of the potential side effects of therapy and lack of anticipatory guidance on toxicity management. Some participants held the misconception that chemotherapy killed patients. They also described chemotherapy as being painful, which they perceived as a combination of physical pain—myalgias and fatigue from treatment—and the psychological toll related to treatment side effects. Furthermore, some of the side effects such as alopecia or skin discoloration were drivers of stigma experienced by women undergoing treatment.

*Some say that it [chemotherapy] can kill one’s brain and then you die, there is just a lot of things that are said about it and that is what made me scared because I was thinking I might actually die on that day. [Low fidelity, PWH]*

*…[nails] became black and my hands also darkened, my face looked like I had burnt. So that being the case means that you become uncomfortable mixing with other people. You can’t exactly go into the community with dark hands and a face that looks like it was burnt. [Low fidelity, HIV-]*

#### Acceptance and De-stigmatization

Participants identified multiple subthemes of “acceptance”, as facilitators for adhering to timely receipt of therapy and completing the prescribed cancer treatment plan. Social acceptance was identified by participants as a pre-requisite for self-acceptance as a “woman” despite their diagnosis and treatment effects. For instance, women emphasized the need to be accepted as women even after breast loss from a mastectomy. Additionally, this form of social acceptance was important for absolving the participant of shame, guilt or blame that they were responsible for their cancer diagnosis. There was also historical reference to tumors as “phologolo” in Setswana, which translates to animal or wild beast. Subsequently, for participants in whom this reference was relevant, there was a necessary mindset shift of accepting themselves as having a “human” disease and normalizing breast cancer like other “human” diseases, such as diabetes or hypertension. There was a clear emphasis on rejecting the notion that cancer was an animal disease; participants cited examples explaining the importance of accepting their diagnosis was not an animal disease like foot and mouth disease of cattle. The third subtheme was acceptance of a biomedical approach to cancer treatment.

*They (children) are asking why our mother had two breasts and now she has one but I explained to them that that’s how they are going to see me from now on and that they should accept me so that I may also be free. Only when they have accepted me will I be free. [High fidelity, PWH]*

*I was told that I have breast cancer and that I was still the same human being… He asked me to open my heart to everything such as radiation and removing the breast that I should accept whatever help I’m given. I said to him, doctor I have heard you and I accept everything. [Low fidelity, HIV-]*

#### Knowledge of Treatment and Toxicity

Participants identified counseling on the curative intent of treatment, potential side effects, timing and anticipated management of toxicity as important facilitators that promoted treatment completion. Participants identified cancer care providers, including physicians, and peer survivors as credible sources of this information.

*I lost my hair but the doctors had already told me. I lost all the hair in my body. At the time I had plaited my hair, but it all fell off, even in my private parts I lost hair, my eyebrows too… I was aware that I was in the process of being cured so I was okay. [High fidelity, PWH]*

### Social/Subjective Norms: Barriers and Facilitators

#### Community-Level Stigma

Participants identified variable community-level stigmatizing attitudes, including discrimination and negative attitudes toward patients with cancer and in some cases their family members, that contributed to individualized stigma experiences and internalized stigma. They also identified stigmatizing attitudes from other family members as barriers to fidelity.

*When people hear that you have cancer they start discriminating against you. They look at you in an unpleasant way… some people have this tendency of saying nasty things to children… “we heard her mother has breast cancer”. Now such things are the reason why my children don’t want me to go public, my husband also doesn’t want me to go public because of things said by Batswana. [High fidelity, HIV-]*

*I told him [husband] over the phone what the results were [cancer diagnosis] and when I got home I did not find him home… He switched off his phone from there on and up to today I have no idea where he is. [Low fidelity, PWH]*

#### Lack of Cancer Awareness

Participants identified lack of awareness and misinformation about cancer as barriers to obtaining reliable information about cancer and early engagement in cancer care. Some participants also stated that these misconceptions led patients with cancer to consider alternative therapies prior to guideline-concordant therapy.

*sometimes you hear some saying chemo makes the disease worse and contemplating going to traditional doctors where they feel they will get healed. [Low fidelity, PWH]*

#### Trust in the Care Team and Social Support

Participants identified trust in the health care team and social support as facilitators associated with treatment fidelity. Trust was usually built on counseling and support provided by the care team. Participants valued healthcare teams that treated them with dignity and respect. The participants also identified social support from religious/spiritual groups and family members, especially from male partners regarding decisions about mastectomies, as facilitators. Some participants also highlighted peer support from other survivors as an important facilitator for participants during treatment. This support was gained through informal referrals to other survivors and most commonly a text message network called “Fighters Group”, made up of other Batswana cancer survivors.

*It’s the fact that on the very first day when I started chemo, they [the care team] welcomed me with open arms… You took me in and made me feel very welcomed. So even when I go for check-up, the way the nurses and doctors welcomed me at Oncology it made me very happy. Whenever I went for chemo, I went there feeling so active and excited knowing that I’m going to meet the lovely staff that will treat me like an honorable person. When I arrived they would usually call me “Mma X”. [High fidelity, PWH]*

*In fact, before I began treatment a friend of mine referred me to see one lady who had done all the treatment, she had done chemo and radiation and was now on medication. When I saw how fit she was, I was encouraged to also start. [Low fidelity, PWH]*

### Perceived Behavior Control: Barriers and Facilitators

#### Social Determinants of Health

Participants reported social determinants of health (SDOH) factors that negatively influenced their ability to receive and complete timely treatment. These included financial toxicity related to transportation costs for participants who lived far from Gaborone, food insecurity, job insecurity, and difficulty scheduling oncology appointments especially radiation oncology visits. For participants on treatment during the COVID-19 pandemic, these barriers were amplified by lockdown and public transportation shutdown.

*Taking treatment has been a challenge for me since I stay very far looking at transport from “X village” to Gaborone [~450 miles]; it is far and expensive. When I get to Gaborone, I need to find accommodation, but I just forced myself to leave here and sleep in Gaborone and when I’m through with chemo I get on the next bus and go so that if there are any undesirable side effects that I can already be home. [High fidelity, PWH]*

*Now there is an added challenge because of corona [COVID pandemic], imagine you were supposed to go for your check up soon and then just like that there is lockdown without any warning. They don’t even cater for a patient who was supposed to receive chemo, and if you don’t have a car and you are from far away, it becomes very difficult. [Low fidelity, PWH]*

#### Health System Barriers

Participants noted that health system barriers impeded their ability to adhere to recommended treatment schedules. These barriers included lack of adequate counseling about their cancer diagnosis and treatment modalities, lack of appointment reminders and follow-up by the care team, and delays due to cancer drug stockouts.

*I was dealing with so much fear and anxiety wondering if I will still be there tomorrow. So somewhere somehow I began to question if I have really received adequate counselling to prepare me for something as dreadful as this and I realized that there was no counselling…my doctor ended up telling me that the focus was to save my life, they were moving with so much speed given the circumstance around the situation and they thought counselling was really secondary to what they wanted to focus on... [High fidelity, PWH]*

*the time [I missed treatment] was when I was supposed to get my last chemo treatment and there was no medicine in Marina. [Low fidelity, HIV-]*

#### Increased Access to Treatment Facility

Participants identified geographical accessibility and ease of scheduling appointments as important facilitators associated with fidelity to treatment. For most, geographical accessibility was described as living in close proximity to the treatment facility in Gaborone or having resources to stay in Gaborone on the day they were scheduled for cancer treatment.

*Also Kanye is not that far, I cannot fail to get fifty pula to transport me from here to Gaborone. That is one thing that has made life easy, the fact that I don’t stay too far from Gaborone. [Low fidelity, PWH]*

#### High Self-efficacy and Advocate Role

Participants who experienced fewer environmental constraints or health system barriers had high self-efficacy. Participants also highlighted that they felt empowered and motivated if they perceived themselves as cancer survivors and advocates for other newly diagnosed cancer patients. Self-motivation also manifested as resiliency in the face of stigma.

*I have never missed an appointment because I don’t want to do injustice to myself which will force me to blame the doctors afterwards…Honestly my dear I want life that is why I follow what the doctors have told me. When I don’t have money I go and borrow then I pay it back when I’m back from the check-up. [High fidelity, HIV-]*

*I would share with other patients voluntarily and show them this [mastectomy] scar. I would tell them that breast cancer is curable and they should use me as an example. [High fidelity, PWH]*

### HIV-Specific Barriers and Facilitators

Among PWH there were unique barriers including intersectional stigma of both HIV and breast cancer. Participants also identified concerns about increased therapy-related toxicity because of administration of both cancer-directed therapy and HIV treatment. Finally, PWH identified additional barriers related to parallel care systems for both HIV and cancer and challenges coordinating appointments for both.

*The challenges of HIV and cancer are… these two kill, that on its own makes one think that they are definitely going to die when they are diagnosed with HIV and cancer. Some people automatically count themselves out from the people who will live for long.” [Low fidelity, PWH]*

*…it’s usually as if cancer treatment overpowers HIV treatment because it weakens your immune system very much such that you can die if you are weak. [Low fidelity, PWH]*

*When you are in Marina [oncology department] and you tell them that you were also due at another facility for [HIV] blood tests, it becomes very difficult because they have their own schedule and own patients and how many they can take in a day. [Low fidelity, PWH]*

PWH identified having integrated cancer-directed and HIV care or a simplification of their HIV and cancer regimens as facilitators associated with treatment fidelity. Furthermore, PWH felt empowered about management of their cancer because of prior success in managing their HIV.

*The things that helped me on HIV treatment that made the other [cancer] treatment easier was the fact that I already knew when I was supposed to go for my next appointment for both so I was already used to having a reminder on one side and I did the same on my phone so that I do not miss the other treatment. [Low fidelity, PWH]*

*I will be going for [cancer] treatment on a certain date if I discovered that there was a clash [scheduling conflict]… they will give me HIV treatment right there as well. [Low fidelity, PWH]*

## Discussion

This study is one of few studies evaluating patient factors and SDOH associated with adherence to guideline-concordant cancer therapy among patients with breast cancer, including PWH in Botswana. The study identified key areas of SDOH that potentially impact adherence to guideline recommended breast cancer therapy including multiple aspects of stigma, financial toxicity, food insecurity, access challenges to cancer care facility and lack of knowledge about treatment, and anticipated side effects. Participants also identified health system barriers that included inadequate patient counseling, health system delays and difficulty with appointment scheduling. Importantly, our study identified additional treatment barriers for PWH, including intersectional stigma, increased toxicity of cancer-directed therapy while concurrently on antiretroviral therapy and challenges with navigating parallel health care systems for breast cancer and HIV care.

“Acceptance and de-stigmatization” were important facilitators reported by participants. Additional facilitators included trust in the care team and social support, which importantly included peer support from other survivors. Other facilitators were adequate knowledge, high self-efficacy and perceived role as a survivor advocate. Finally, in PWH, being able to integrate care or simplify treatment regimens for both cancer and HIV, promoted adherence to cancer treatment.

These study results must be considered in light of several limitations. This study was limited to women with breast cancer which may limit generalizability to other cancer types. However, this distribution of participants is representative of the distribution of PWH and late-stage breast cancer presentation in Botswana and other countries in SSA. Furthermore, breast cancer experience and recommended surgical interventions are unique to these group of patients with cancer and therefore some aspects of social stigma identified in relation to breast cancer surgery and risk of infertility are not generalizable to the experience of other patients with cancer.^47^ Finally, stigma is in part driven by social norms which are specific to cultural contexts, and therefore experiences of breast cancer stigma or intersectional stigma in this population may not be globally generalizable.^31^

In spite of these limitations, the study had several strengths. We used a deviance sampling of participants with low and high fidelity and purposively sampled PWH to potentially capture themes exclusive to patients with either high or low fidelity. Although we identified common facilitators and barriers in both fidelity groups, we identified unique challenges and facilitators for PWH. This sampling and analytic approach will ensure that future interventions are tailored to specific populations, for example, PWH, to avoid widening disparities in breast cancer outcomes. The use of culturally-, language-, and gender-matched interviewers from Botswana ensured that emerging themes were adequately probed in-depth.^44^ The multidisciplinary team of researchers in HIV and investigators from Botswana was an added strength and shed light on cultural nuances and historical relevance of some of the themes identified.

Prior studies have evaluated contextual barriers and facilitators that influence patient delays and breast cancer and cervical cancer screening uptake and although similar themes such as knowledge, access, and social norms have been identified,^48, 49^ this study is unique in identifying barriers and potential opportunities for improving timely treatment initiation and completion. Other studies that have examined treatment abandonment and treatment completion highlight the prevalence and importance of the problem.^50^ For instance, a multi-country prospective cohort analysis showed variation in treatment initiation, with 32% of women in Nigeria with curative breast cancer, stages I–III, not initiating curative treatment within a year of diagnosis.^51^ In a recent study, health system factors, such as improving cancer care access and comprehensive cancer care coverage, were found to be associated with high-quality treatment delivery.^51^However, the findings from our study indicate that understanding patient-level perspectives are important for tailoring interventions to address adherence to breast cancer treatment even when national guidelines and comprehensive cancer care coverage is available for patients in-country.

Breast cancer and HIV intersectional stigma is an important but relatively unexplored area of study. There is emerging data from other parts of sub-Saharan Africa which emphasize the important barrier of stigma that influences care engagement and treatment adherence.^8,52,53^ Additionally, although the body of work around HIV stigma in Botswana is extensive, there are few studies specifically addressing intersectionality of HIV and breast cancer stigma.^54-56^ Interventions that have been successful in addressing HIV stigma in Botswana may be tailored and tested among breast cancer PWH and those without HIV, especially leveraging the idea of womanhood and what’s important in the Botswana cultural context.^57^

The theme of acceptance has been previously identified as an important facilitator among breast cancer patients in Uganda,^53^ and in women with HIV in Botswana who identified the need for self-acceptance as a means of contesting associated HIV stigma-related to promiscuity and preserving what it means to be a woman in Setswana culture.^58^ Similarly, breast cancer is unique in that having a mastectomy or risk of infertility for patients on cytotoxic therapy or ovarian suppression, poses a threat to the perception of what it means to be a woman in Setswana culture. Participants made references to a distortion of their appearances as being “disabled” or disfigured following a mastectomy, further emphasizing this theme. Previous studies have identified similar themes of stigma associated with a mastectomy in SSA.^8^ Although prior studies identified that members from the healthcare team and religious groups were most effective in the process of promoting self-acceptance,^53,58^ our study identified social acceptance from the larger community and nuclear family as being integral to the process of self-acceptance. Realignment with curative intent of treatment, usually if supported by the participant’s social support structure led to acceptance of diagnosis, treatment, and self-validation of their womanhood in society.

We plan to evaluate the prevalence of these themes by developing quantitative tools to capture qualitative themes and assess the association among the quantitatively assessed measures and treatment fidelity. The results will inform prioritization, design, and implementation of different interventions to promote fidelity. The study findings highlight potential areas where multilevel interventions that address stigma and distress may be designed to screen for and provide linkage to resources, such as psychosocial services. Additionally, we identified themes of resilience in the face of stigma, consistent with empowerment and perceived role as advocate, as facilitators of treatment. Social scientists postulate that resilience is important for overcoming stigma,^59^ which could be leveraged in designing interventions, such as peer navigation, community health worker interventions and survivor support groups, to promote cancer treatment fidelity. Additionally, previous studies have shown religious/spiritual support as an important facilitator^8,53^ that has been leveraged by patients with breast cancer in informal ways. This may be tested more formally in future interventions to facilitate treatment adherence.^60^

## Conclusion

We identified stigma and other multilevel modifiable patient and health system factors associated with treatment fidelity to guideline-concordant breast cancer treatment. The facilitators provide opportunities for leveraging existing strengths within the Botswana context to design implementation strategies to increase treatment fidelity to guideline-concordant breast cancer therapy. However, PWH experienced unique barriers, suggesting that interventions to address treatment fidelity may need to be tailored to individual circumstances such as comorbidities.

## Funding

This study was funded by a Fogarty International Center, NIH K01 Award (PI: Martei).

## Data Availability

The data underlying this article cannot be shared publicly due to for the privacy of individuals that participated in the study. The data will be shared on reasonable request to the corresponding author and will need to be reviewed by the Botswana IRB.
